# Care pathways and anorectal evaluation for obstetric anal sphincter injury‐related incontinence: A UK survey of obstetricians

**DOI:** 10.1111/codi.70140

**Published:** 2025-06-27

**Authors:** N. Elsaid, G. P. Thomas, S. Dutta, R. J. Fernando, E. V. Carrington, C. J. Vaizey

**Affiliations:** ^1^ St Mark's the National Bowel Hospital London UK; ^2^ Imperial College London UK; ^3^ St Mary's Hospital London UK

**Keywords:** anorectal function, care pathways, follow up, incontinence, obstetric anal sphincter injury, obstetrician, referral, surgeon, urogynaecologist

## Abstract

**Aim:**

To report on national clinical practice in relation to the post‐partum management of patients with obstetric anal sphincter injury (OASI)‐related incontinence in the UK.

**Method:**

This was a cross‐sectional, observational study of maternity units in the National Health Service (NHS). Data were collected using a survey that was distributed, via the British Society of Urogynaecologists (BSUG), Royal College of Obstetricians & Gynaecologists (RCOG) and the NHS England email directory, to consultant obstetricians and urogynaecologists involved in the post‐partum care of patients with OASI. A descriptive, thematic analysis of the data was performed.

**Results:**

One hundred and twenty‐six responses were included in the final analysis (estimated response rate~2.5%). The majority of respondents routinely conducted clinical and rectal examinations at the post‐partum clinic visit (81.7% and 57.6%, respectively) but they were less likely to use an objective screening tool for incontinence (36.5%). Respondents were more likely to refer patients for anorectal studies if they were symptomatic (72.6%) rather than asymptomatic (34.2%); the diagnostic modality of choice was endoanal ultrasound (70%) rather than manometry (0%). Almost 80% of respondents discharged asymptomatic patients within 3 months. All respondents referred symptomatic patients for physiotherapy; 87% were seen within 6 weeks. Although 70% would discuss complicated cases at a multidisciplinary team meeting (MDT), there was a wide variation in which speciality would follow up the patient.

**Conclusion:**

This study demonstrates variability in clinical practice that does not entirely appear to be evidence driven. A nationally endorsed pathway, embedded within Perinatal Pelvic Health Services, could standardize access to expertise and enable benchmarking. A prospective multicentre audit is recommended to compare maternal functional outcomes in units that use these standardized pathways versus those that do not.

**What does this paper add to the literature?**

This study highlights national variation in post‐partum obstetric anal sphincter injury (OASI) care and the need for standardized pathways. It uniquely explores how colleagues from sister specialities – urogynaecology and obstetrics – manage OASI‐related incontinence, emphasizing the value of multidisciplinary collaboration to optimize outcomes and ensure equitable, evidence‐based care across the UK.

## INTRODUCTION

Perineal tears are a recognized complication of vaginal delivery and are classified into four categories, according to severity [1, 2]. Third‐ and fourth‐degree tears, involving the anorectal sphincter complex, are known as obstetric anal sphincter injuries (OASIs) and are associated with considerable maternal morbidity [3]. Symptoms may occur in 30%–40% of affected women, and include faecal and urinary incontinence, prolapse and perineal pain [4, 5]. Obstetric anal sphincter injuries are the most common cause of perineal trauma and faecal incontinence in women and have been identified as a significant cause of litigation in obstetrics [3, 6, 7, 8]. Following reports that the rate of an OASI had tripled between 2000 and 2012, the Royal College of Obstetricians & Gynaecologists (RCOG) OASI Care Bundle was introduced in a bid to reduce the incidence of OASIs and primarily focused on interventions in the antenatal period [9, 10, 11]. However, even with the best efforts of care, OASIs cannot be eliminated. Therefore, focus should also be placed on the effective post‐partum management of patients with an OASI, to reduce the physical, psychological, socioeconomic and financial burden of such injuries.

The RCOG states that patients with an OASI must be followed up 6–12 weeks post‐partum and that a referral to a colorectal specialist should be considered in those who are symptomatic of incontinence [3, 7]. However, there appears to be a large variation in the method of follow up between units in the UK. In the absence of a standardized national pathway, the provision of healthcare appears to rely on the availability of resources, a surgeon's discretion and ultimately a ‘postcode lottery’. While some trusts may have the necessary provisions in place to support mothers with OASIs, in other areas, women may lack the necessary support.

The purpose of this study was to report on national clinical practice in relation to the post‐partum investigation, management and follow up of patients with OASI‐related incontinence in the UK. This is particularly important as afflicted individuals may not readily volunteer their symptoms and struggles and need to be safeguarded by the presence of robust care pathways that ensure adequate follow up and care provision. Owing to the stigma surrounding the sequelae of OASIs, women may be reluctant to seek help, leading to a silent affliction and under‐reporting of symptoms [12, 13, 14, 15, 16]. Furthermore, those who are initially asymptomatic may develop symptoms later in life secondary to advancing age, the impact of hormonal changes on pelvic floor function and the added impact of further deliveries [17, 18]. Anal incontinence may also persist for several years following vaginal delivery [19]. Long‐term accessibility to evidence‐based therapies and support is therefore advocated for women with an OASI.

## METHOD

A cross‐sectional, observational study was performed. Ethical approval was obtained from the London Social Care Research Ethics Committee, Health Research Authority and Health and Care Research Wales in January 2024 (study ref. 23/IEC08/0046). The study was registered on ClinicalTrials.gov, Protocol Registration and Results System (PRS) (NCT06143072).

A survey was developed using the Qualtrics survey tool. The questions were developed in line with the evidence‐based RCOG Green‐top guideline No. 29, entitled ‘Third‐ and Fourth‐degree Perineal Tears, Management’. A review of the literature was also undertaken by the research group, and a clinician involvement group (comprising urogynaecologists, colorectal surgeons and biofeedback nurse specialists) was consulted to ensure the questionnaire was comprehensive and clinically appropriate. The validity of the questionnaire was assessed using a clinical focus group of 15 obstetricians.

Obstetricians and urogynaecologists, who were on the General Medical Council specialist register and were practising in the UK at the time of the study, were invited to participate if they were involved in the care of patients with OASIs post‐partum. The survey was emailed to members of the British Society of Urogynaecologists (BSUG) and the RCOG. Using National Health Service (NHS) digital workforce statistics, the authors identified approximately 4800 consultant obstetricians and gynaecologists and 290 subspecialist urogynaecologists at the time of survey. The RCOG confirmed that the survey was sent to around 5000 UK members. Both societies sent out a reminder to members several weeks after initial dissemination. The survey was also distributed via social media platforms, including LinkedIn and Twitter as well as the social software chat group, WhatsApp. Maternity Units were also identified using the NHS England hospital directory and individual consultants were emailed via the NHS England email directory. A snowball sampling method was also used whereby consultants were encouraged to forward the survey to their colleagues, to encourage wider participation recruitment. The survey was distributed over a 4‐month period from 1st April to 31st July 2024.

Consent was implied after reading the participant information sheet and voluntarily completing the survey. Remuneration was not offered for participation in this study. Adaptive questioning was used, with a minimum of 19 and a maximum of 22 items. The question order was identical for all respondents. Where a question was left blank, a response of ‘did not answer’ was recorded. Completion time was around 5 min. To maintain anonymity, neither personal identifiable data nor the respondent's IP address was recorded. Descriptive analysis of the quantitative data and thematic analysis of the qualitative data were performed.

The Checklist for Reporting Results of Internet E‐Surveys (CHERRIES) tool was followed to ensure rigorous reporting of the survey.

## RESULTS

In total, 166 responses were recorded. Forty of the responses were excluded from the dataset as all questions were left blank in these submissions. One hundred and twenty‐six responses were included in the final analysis. This corresponds to an overall response rate of ~2.5%.

**FIGURE 1:**
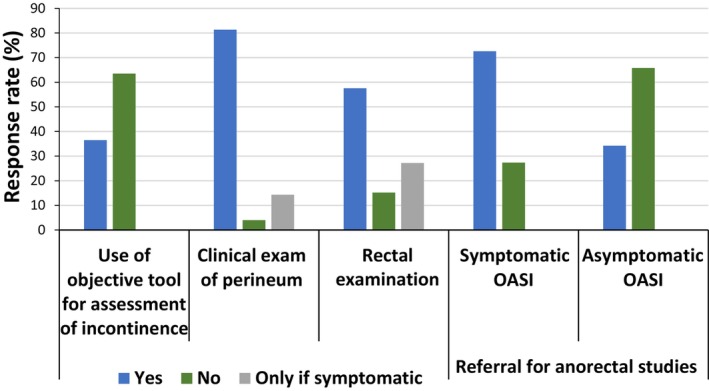
Post‐partum evaluation of patients with obstetric anal sphincter injury (OASI).

**FIGURE 2:**
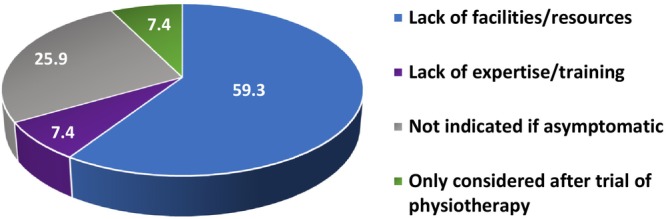
Reasons given for not referring patients with obstetric anal sphincter injury (OASI) for anorectal studies. The numbers represent percentages (%).

Responses were obtained from all regions across the UK, with the greatest number recorded from London (27.8%) (Table S1). The majority of respondents stated that they routinely conducted clinical and rectal examinations at the post‐partum clinic visit (81.7% and 57.6%, respectively) but that they were less likely to use an objective screening tool for incontinence (36.5%). Respondents were more likely to refer patients for anorectal studies if they were symptomatic (72.6%) rather than asymptomatic (34.2%) (Figure 1), and the diagnostic modality of choice was endoanal ultrasound (70%) rather than manometry (0%) (Figure S1). Various reasons were given for not referring patients for anorectal studies, including lack of resources and training (Figure 2).

**FIGURE 3:**
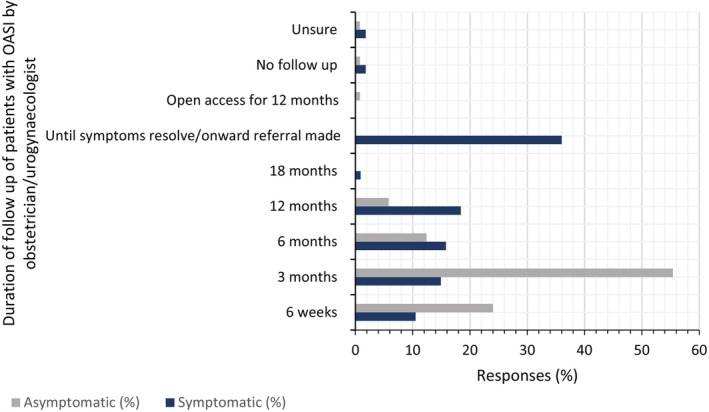
Duration of follow up of patients with obstetric anal sphincter injury (OASI) by obstetrician/urogynaecologist.

Almost 80% of respondents discharged asymptomatic patients within 3 months (Figure 3, Table S3). All respondents referred symptomatic patients for physiotherapy and 87% of these patients were seen by a physiotherapist within 6 weeks (Table S4, Figure S2).

**FIGURE 4:**
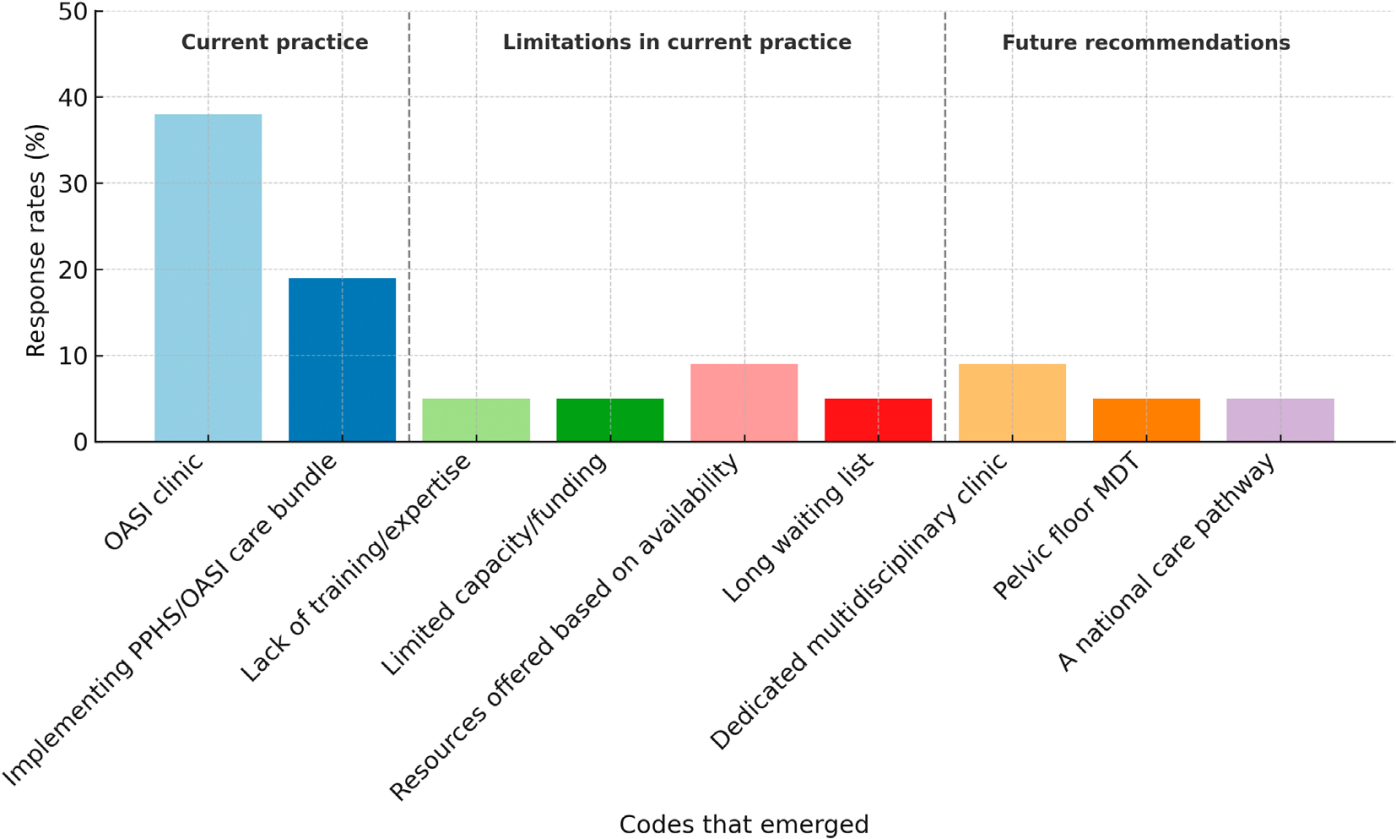
Themes that emerged when respondents were asked to make additional comments. MDT, multidisciplinary team, OASI, obstetric anal sphincter injury, PPHS, Perinatal Pelvic Health Service.

When asked to make additional comments in relation to this study, the respondents highlighted limitations in current practice (lack of expertise and training in conducting anorectal studies, limited funding and long waiting lists for investigations, therapies and referral to specialist), as well as recommendations for future practice (a collaborative approach to patient care, multidisciplinary clinics and meetings, and the development of a national pathway) (Figure 4).

## DISCUSSION

### Main findings

#### Pelvic floor physiotherapy

Pelvic floor muscle training aims to strengthen the anal sphincter and has been shown to improve incontinence symptoms [20, 21, 22]. Perinatal Pelvic Health Services have recently emerged in a bid to improve maternal accessibility to physiotherapy and multidisciplinary pelvic health services in the post‐partum period [23]. In this survey, all respondents referred their symptomatic patients for physiotherapy and 83% stated that their patients were seen within 6 weeks post‐partum (Table S4, Figure S2). There is evidence to suggest that early intervention with physiotherapy, in the first 4 weeks post‐partum, leads to improved maternal outcomes [24].

#### History and examination

This study confirmed substantial variability in post‐partum OASI follow‐up and highlights under‐utilization of symptom scoring tools and anorectal physiology testing. Moreover, this study identified that 64% of respondents did not use an objective tool when screening for incontinence (Figure 1, Table S2). History‐taking alone may be inadequate and subject to clinician bias [25]. Furthermore, women who deny frank faecal incontinence may still experience incontinence to flatus. Paradoxically, flatal incontinence has been shown to have a greater deleterious impact on quality of life than faecal incontinence [26].

In total, 82% of respondents routinely offered a clinical examination of the perineum at the post‐partum check. A smaller proportion of respondents (58%) would offer patients a rectal examination (Figure 1, Table S2). Both the National Institute of Health and Care Excellence (NICE) guideline No. 235 and the OASI care bundle guidance state that all women who have given birth vaginally should be offered a rectal examination, even when the perineum appears intact, to exclude occult injury to the genital tract, such as buttonhole tears [10, 27]. It should be noted, however, that clinical and rectal digital examination alone may not be sufficient to identify occult sphincter defects and therefore referral for anorectal studies and endoanal ultrasound is still necessary [28]. The presence of residual sphincter defects following a primary repair has been associated with a greater risk of long‐term complications [5, 29, 30, 31].

#### Diagnostic evaluation

In total, 27.4% of respondents would not refer symptomatic patients for anorectal studies. Data suggest that objective evaluation of anorectal anatomy and function can influence clinical decision making and the subsequent management of patients with incontinence, including identifying appropriate candidates for surgery [32, 33].

Although endoanal ultrasound was the preferred diagnostic modality among 70% of respondents in this study, the current literature suggests that both endoanal ultrasound and anal manometry provide complementary diagnostic information and may be best utilized in combination. Endoanal ultrasound offers direct anatomical visualization of sphincter integrity and has reported sensitivities ranging from 80% to 100% and specificities between 70% and 90% for detecting sphincter defects when compared with surgical findings [28]. However, endoanal ultrasound is less sensitive than manometry in identifying functional abnormalities in the absence of structural damage. By contrast, anal manometry, which assesses functional sphincter pressures, has been shown to identify abnormalities in up to 75% of asymptomatic women with a prior OASI [34]. Badri et al. also reported that anal manometry detected more cases of dysfunction than endoanal ultrasound alone, reinforcing its value in functional assessment [34, 35]. Despite this, no respondents in this survey selected anal manometry as a standalone test, and only 30% referred patients for both anal manometry and endoanal ultrasound (Table S2, Figure S1). The variation in practice may be attributed to limited resources, insufficient access to trained personnel, and institutional constraints, rather than adherence to evidence‐based protocols. Nonetheless, the authors acknowledge that a much smaller sample of consultants had answered this question (*n* = 20) and the findings, therefore, must be interpreted judiciously.

Furthermore, anal manometry may serve as a predictive tool in identifying women at increased risk of developing future anal incontinence. Roos et al. [28] demonstrated that reduced anal sphincter pressures, particularly squeeze pressures, were significantly associated with symptom severity and could predict the development of incontinence symptoms, even in previously asymptomatic women. This highlights the potential role of manometry, not only in diagnosis but also in predicting symptom development and informing long‐term management strategies for women with prior OASI.

#### Implications for future birth planning

Almost all the respondents (96.6%) in this study reported that they would counsel their patients regarding future mode of delivery. However, only 34.2% would refer asymptomatic patients for anorectal studies compared with 72.6% if the patient was symptomatic (Figure 1, Table S2). Of those who did not refer asymptomatic patients for anorectal studies, 25.9% believed that it was not indicated (Figure 2). A prospective study demonstrated that 75% of asymptomatic women with an OASI had at least one abnormal anorectal study and evidence‐based guidance states that asymptomatic women should be offered an elective Caesarean section in the event of ‘abnormal anorectal manometric pressures and/or ultrasonographic sphincter defects’ [3, 34, 36]. Therefore, it is unclear from this study whether asymptomatic women were being counselled correctly. As third‐ and fourth‐degree tears are more common in primiparous women (6.1%, compared with 1.7% in multiparous women), evidence‐based counselling regarding future mode of delivery is crucial for these women who may wish to have more children [36, 37, 38]. The importance of this has also been highlighted by the Montgomery legal ruling in the UK [39].

#### Hospital policies and follow‐up duration

Twenty three percent of respondents stated that their Trust did not have a policy for the management of OASI‐related incontinence (Table S5). Research has demonstrated the positive effects of implementing a protocol for the management of OASIs and the RCOG advises all units to have a clear policy in place [3, 40].

This study demonstrated that the duration of follow up for symptomatic women was unlikely to exceed 12 months. There is evidence to suggest that 60%–80% of women are asymptomatic at 12 months [3]. Reid et al. [41] showed that 10% of asymptomatic women with sphincter defects on ultrasound eventually developed symptoms within 3 years post‐partum. Almost 80% of respondents would discharge asymptomatic patients 6–12 weeks post‐partum (Figure 3, Table S3). Provisions should be in place to ensure ease of access to services if this was subsequently required following discharge. Eccles et al. [42] highlighted the difficulties women face when attempting to seek help for incontinence in the primary care setting. Open‐access follow‐up appointments (patient‐initiated follow up), as highlighted by a respondent in this study (Table S3), can empower women to reach the services they need, when they require it [43].

### Strengths and limitations

The strengths of this study include nationwide sampling and alignment with evidence‐based guidance, and the limitations comprise possible responder bias and self‐reported practice. It was not possible to calculate the number of consultant obstetricians and urogynaecologists specializing in OASI care who were currently practising in the UK because the available data does not distinguish between clinicians with different subspeciality interests.

Although the number of eligible obstetricians that this survey had reached was estimated, many obstetricians replied to the researcher that they were not involved in the care of OASI patients and therefore a precise response rate could not be confirmed. This illustrates that it is not well defined, within the obstetrics community, who is ultimately responsible for the care of patients with OASI.

Despite dissemination of the survey to the members of two major obstetric societies and utilization of various platforms to optimize recruitment for the study, the overall response rate was low. This may be attributed to several factors, including survey fatigue among clinicians, time constraints in a demanding clinical speciality, limited engagement with digital communication channels, or perceived irrelevance of the topic by some recipients. Nevertheless, the sample size is considered comparable to that of similar electronic surveys targeting senior clinicians [37]. Further research should investigate regional disparities owing to the small sample size of some of the individual regions.

### Interpretation

If conservative measures for the management of incontinence fail, other treatment options may need to be considered, including percutaneous posterior tibial and sacral nerve stimulation, secondary sphincteroplasty and stoma formation. Specialist pelvic floor input may be required; however, it is not clear from this study whether a robust referral pathway exists to facilitate this option. Although 70% of respondents would discuss complicated cases at an MDT, there was a wide variation in the assessment, investigation and referral pathways for patients with OASI‐related incontinence (Table S5).

## CONCLUSION

The management of OASI‐related incontinence is multifaceted, requiring input from various specialists, including allied healthcare professionals. The development of dedicated multidisciplinary perineal clinics, including both urogynaecologists and pelvic floor surgeons, can promote a holistic approach to patient care. A nationally endorsed pathway, embedded within Perinatal Pelvic Health Services, could standardize access to expertise and enable benchmarking. A prospective multicentre audit is recommended to compare maternal functional outcomes in units that use these standardized pathways versus those that do not.

## AUTHOR CONTRIBUTIONS

**N. Elsaid:** Conceptualization; methodology; data curation; investigation; formal analysis; writing – original draft. **G. P. Thomas:** Conceptualization; methodology; supervision; writing – review and editing. **S. Dutta:** Conceptualization; methodology; writing – review and editing. **R. J. Fernando:** Conceptualization; methodology; writing – review and editing. **E. V. Carrington:** Conceptualization; methodology; supervision; writing – review and editing. **C. J. Vaizey:** Conceptualization; methodology; supervision; writing – review and editing.

## FUNDING INFORMATION

This research received no specific grant from any funding agency in the public, commercial or not‐for‐profit sectors.

## CONFLICT OF INTEREST STATEMENT

None declared.

## ETHICS STATEMENT

Ethical approval was obtained from the London Social Care Research Ethics Committee, Health Research Authority and Health and Care Research Wales in January 2024 (study ref. 23/IEC08/0046). Confirmation of capacity and capability was successfully attained at London Northwest University Healthcare Trust prior to study commencement (study sponsor).

## Supporting information


Appendix S1.

## Data Availability

The data that support the findings of this study are available on request from the corresponding author. The data are not publicly available due to privacy or ethical restrictions.
