# Supplementary material for: Care pathways and anorectal evaluation for obstetric anal sphincter injury‐related incontinence: A UK survey of obstetricians
Source: Colorectal Dis. 2025 Jun 27;27(7):e70140. doi: 10.1111/codi.70140 (PMC12205113; doi:10.1111/codi.70140)
Supplement: Supplementary file 1 — Appendix S1. [file CODI-27-0-s001.docx]

**Supplementary Figures and Tables:**

**Supplementary table 1: Demographics**

| **Region currently practising in** | **n (%)** |
| --- | --- |
|  |  |
| East of England | 18 (14.3) |
| London | 35 (27.8) |
| Midlands | 19 (15.1) |
| North East and Yorkshire | 13 (10.3) |
| North West | 10 (7.9) |
| South East | 10 (7.9) |
| South West | 13 (10.3) |
| Scotland | 4 (3.2) |
| Wales | 2 (1.6) |
| Northern Ireland | 1 (0.8) |
| Crown Dependencies | 1 (0.8) |
| Did not respond | 0 |

**Supplementary table 2: Post-partum evaluation of OASI patients**

| **Use of objective screening tool for assessment of incontinence** | n (%) |
| --- | --- |
| Yes | 46 (36.5) |
| No | 80 (63.5) |
| Did not respond | 0 |
|  |  |
| **Clinical examination of the perineum** |  |
| Yes | 103 (81.7) |
| No | 5 (4.0) |
| Only if symptomatic | 18 (14.3) |
| Did not respond | 0 |
|  |  |
| **Rectal examination** |  |
| Yes | 72 (57.6) |
| No | 19 (15.2) |
| Only if symptomatic | 34 (27.2) |
| Did not respond | 1 |
|  |  |
| **Referral for anorectal studies** |  |
| Symptomatic OASI |  |
| Yes | 85 (72.6) |
| No | 32 (27.4) |
| Did not respond | 9 |
| Asymptomatic OASI |  |
| Yes | 40 (34.2) |
| No | 77 (65.8) |
| Did not respond | 9 |
|  |  |
| **Which anorectal studies are OASI patients referred for?** |  |
| EAUS only | 14 (70) |
| Manometry only | 0 (0) |
| Both EAUS and Manometry | 6 (30) |
|  |  |
| **Counselling of patients regarding mode of delivery for future pregnancies** |  |
| Yes | 113 (96.6) |
| No | 4 (3.4) |
| Did not respond | 9 |

**Supplementary Figure 1: Type of anorectal studies OASI patients referred for**

**Supplementary Table 3: Duration of Follow Up of OASI patients by Obstetrician/Urogynaecologist**

| **Symptomatic** | n (%) |
| --- | --- |
| 3 weeks | 1 (0.9) |
| 6 weeks | 11 (9.6) |
| 3 months | 17 (14.9) |
| 6 months | 18 (15.8) |
| 12 months | 21 (18.4) |
| 18 months | 1 (0.9) |
| Until symptoms resolve/onward referral made | 41 (36.0) |
| Discharge to physiotherapy/biofeedback | 2 (1.8) |
| Unsure | 2 (1.8) |
| Did not respond | 12 |
|  |  |
| **Asymptomatic** |  |
| 6 weeks | 29 (24.0) |
| 3 months | 67 (55.4) |
| 4 months | 1 (0.8) |
| 6 months | 14 (11.6) |
| 12 months | 7 (5.8) |
| Other | 2 (1.7) |
| No follow up | 1 |
| Open access for 12 months | 1 |
| Unsure | 1 (0.8) |
| Did not respond | 5 |

**Supplementary Table 4: Referral pathway for patients with OASI-related incontinence**

| **Physiotherapy** | n (%) |
| --- | --- |
| Yes | 112 (100) |
| No | 0 |
| Did not respond | 14 |
|  |  |
| **Time to first review by physiotherapist** |  |
| Immediately post-partum | 35 |
| 6 weeks | 52 |
| 3 months | 11 |
| 6 months | 6 |
| >6 months | 1 |
| Unsure | 4 |

**Supplementary Figure 2: Referral pathway for patients with OASI-related incontinence**

**Supplementary Table 5: Multi-disciplinary management of OASI-related incontinence**

| **Does your hospital have a policy/pathway for the management of post-partum OASI related incontinence?** | **N (%)** |
| --- | --- |
| Yes | 86 (76.8) |
| No | 26 (23.2) |
| Did not respond | 14 |
|  |  |
| **Are patients with complicated or symptomatic OASI discussed at a dedicated MDT?** |  |
| Yes | 78 (70.9) |
| No | 32 (29.1) |
| Did not respond | 16 |
|  |  |
| **How are patients with OASI related incontinence investigated and managed following initial follow up?** |  |
| ‘In house’ management by urogynaecologist only | 25 (22.5) |
| ‘In house’ management by urogynaecologist and pelvic floor surgeon | 48 (43.2) |
| ‘In house’ management by general colorectal surgeon | 10 (9.0) |
| Onward referral to GP | 0 (0) |
| Onward referral to general colorectal surgeon locally | 11 (9.9) |
| Onward referral to pelvic floor specialist at dedicated perineal clinic | 17 (15.3) |
| Did not respond | 15 |
|  |  |
| **How long does it take for patients to be seen by the specialist above?** |  |
| <3 months | 22 (19.8) |
| 3-6 months | 55 (49.5) |
| 6-12 months | 20 (18.0) |
| 12-18 months | 8 (7.2) |
| Unsure | 6 (5.4) |
| Did not respond | 15 |
